# Genome-Wide Identification of the Heterotrimeric G-Protein Gene Family and Its Transcriptional Response to Salt Stress in Foxtail Millet

**DOI:** 10.3390/biology15161339

**Published:** 2026-08-07

**Authors:** Xiuyan Cui, Wei Guo, Ying Han, Yiting Zhang, Shike Zhao, Ling Chen, Wei Zhou, Haigang Wang, Xiang Tian, Junjie Wang

**Affiliations:** 1 Center for Agricultural Genetic Resources Research, Shanxi Agricultural University, Taiyuan 030031, China; 13234759609@163.com (X.C.); gw606283@163.com (W.G.); hy271474846@163.com (Y.H.); 17698896838@163.com (Y.Z.); 16602701286@163.com (S.Z.); chenling832013@163.com (L.C.); nkywhg@126.com (H.W.); 2 College of Agriculture, Shanxi Agricultural University, Jinzhong 030801, China; 3 Shanxi Institute for Functional Food, Shanxi Agricultural University, Taiyuan 030031, China; nx06114@163.com

**Keywords:** foxtail millet, G protein, gene family, salt stress, molecular breeding

## Abstract

Heterotrimeric GTP-binding proteins, commonly known as G proteins, act as core molecular switches in plant signal transduction, governing multiple biological processes including plant growth, development, phytohormone signaling, and abiotic stress adaptation. In this study, a total of 12 G-protein-related genes were identified in foxtail millet, consisting of six Gα subunit-encoding genes, one Gβ subunit-encoding gene, and five Gγ subunit-encoding genes. Gene structure and conserved motif analyses showed that members classified into the same subunit clade possessed highly conserved exon–intron organization and motif distribution, indicating evolutionary structural conservation within each G-protein subfamily. Promoter *Cis*-acting element analysis revealed that all G-protein family genes harbored light-responsive elements, as well as multiple regulatory elements associated with phytohormone signaling and abiotic stress responses. Transcriptome analysis showed that different G-protein genes exhibited distinct expression patterns under salt stress. Notably, *SiαXLG2* exhibited drastically induced expression upon salt stress, which serves as a pivotal candidate gene for salt-stress response in foxtail millet. Collectively, this study provides a systematic characterization of the G-protein gene family and offers candidate gene resources for further elucidating G-protein-mediated salt-stress regulatory mechanisms in foxtail millet.

## Simple Summary

G proteins are ubiquitous in plants and play an important role in many biological functions such as plant growth and development, stress response and so on. However, their specific function in foxtail millet (*Setaria italica* L.) is not clear. In this study, 12 G-protein gene family members were identified in foxtail millet and divided into four categories, and their whole-genome characteristics were analyzed. Transcriptome analysis showed that these members showed different expression patterns under salt-stress treatment at different times. This study increased the understanding of the G-protein gene family and provided valuable genetic resources for foxtail millet breeding.

## 1. Introduction

Soil salinization–alkalization constitutes a major abiotic stress that puts serious constraints on plant growth, development, and productivity [1]. Soil salinity stress is primarily triggered by excess neutral salts (e.g., NaCl and Na_2_SO_4_) in topsoil. Under salt-stress conditions, excessive Na infiltrates plant cells, competitively inhibiting the transmembrane absorption and homeostasis maintenance of essential cations (K and Ca), thereby triggering intracellular ionic toxicity and disrupting the critical Na/K ionic balance, consequently causing developmental delays, reactive oxygen species (ROS) accumulation [2,3,4], membrane system damage, chloroplast structural disintegration, and reduced photosynthetic efficiency [5]. Ultimately, these effects trigger tissue senescence and plant death [6]. A previous study verified that salt stress significantly restricts total biomass accumulation and root system development in crops, further weakening plant salt adaptability [7]. Therefore, elucidating the molecular mechanisms of plant salt–alkali tolerance [8] can enhance stress resistance and facilitate the sustainable utilization of saline–alkaline land resources. This has strategic significance in ensuring agricultural sustainability and ecological security.

Heterotrimeric G proteins are highly conserved and widely distributed in eukaryotes, functioning as core signaling regulators to modulate diverse plant biological processes, including seed germination, seedling growth, plant morphogenesis, stomatal movement, and plant responses to biotic and abiotic stresses [9,10,11]. Canonical heterotrimeric G-protein complexes consist of three distinct subunits with different molecular weights: Gα (40–46 kDa), Gβ (37–44 kDa), and Gγ (6–9 kDa). In vivo, Gβ and Gγ subunits stably form an obligate Gβγ dimer [12]. Structurally, the Gα subunit possesses a Ras-like nucleotide-binding domain and a coiled-coil helical domain [13]. The Gβ subunit features seven conserved WD40 repeat domains, and its N-terminal helical coil domain mediates dimerization with Gγ subunits [14]. Plant Gγ subunits are divided into three structural subtypes. Type A Gγ proteins contain an N-terminal helical domain for Gβ dimerization and a C-terminal CaaX isoprenoid modification motif. Type B Gγ proteins lack the CaaX motif but harbor a C-terminal coiled-coil domain and cysteine-rich domain [15]. As pivotal transmembrane signal transducers, heterotrimeric G proteins constitute an evolutionarily conserved signaling cascade in both plants and animals and have become a research hotspot in plant signal regulatory networks. Upon sensing extracellular environmental cues or hormonal signals, plants activate membrane-localized G-protein-coupled receptors (GPCRs) via specific ligand binding, which triggers GPCR conformational changes. Activated GPCRs further act on GDP-bound inactive G-protein heterotrimers, promoting GDP-GTP exchange on the Gα subunit. GTP-bound Gα subsequently dissociates from the Gβγ dimer and GPCRs and interacts with downstream effectors to trigger intracellular regulatory responses, thereby completing transmembrane signal transduction [16]. Functional genetic studies have verified the critical roles of G-protein subunits in plant growth, development and stress defense. Under abiotic stress, rice *rga1* mutants maintain higher photosynthetic efficiency, optimized root–shoot ratio and improved stomatal conductance under cadmium stress [17]. In contrast, *rgb1* loss-of-function mutants are hypersensitive to salt, cold and drought, while *RGB1* overexpression significantly enhances rice thermotolerance and salt resistance [18]. Genome-wide identification has revealed obvious interspecific differences in the number of members of plant G-protein families. The *Arabidopsis* genome contains eight G-protein genes, including canonical GPA-type and XLG-type Gα subfamily genes, one Gβ gene, and three Gγ genes. A total of 11 G-protein members have been identified in rice, including five Gα, one Gβ and five Gγ genes [19]. The G-protein family in soybean (*Glycine max*) comprises 21 members (13 Gα, four Gβ and four Gγ genes) [20]. Rapeseed (*Brassica napus* L.) has the largest known plant G-protein family, with 28 identified genes, covering eight Gα, seven Gβ and 13 Gγ subunits [21].

Foxtail millet (*Setaria italica* L.), also known as millet, is one of the three domesticated millet species; its cultivation and production are severely threatened by salinity stress [22,23,24,25,26,27]. G-protein families have been extensively characterized in major staple crops, yet systematic identification and functional mining of G proteins remain absent in foxtail millet, and their biological functions require further investigation. Consequently, the present study systematically identified fifteen G-protein-encoding genes from the foxtail millet reference genome. Subsequently, integrated bioinformatics approaches were used to characterize their physicochemical properties, phylogenetic relationships, chromosomal locations, gene structures, conserved motifs, *cis*-regulatory elements, and synteny relationships. Foxtail millet seedlings treated with salt for different durations were sampled for transcriptome sequencing, with emphasis on their expression dynamics under salt–alkali stress. Ultimately, a candidate gene associated with salt resistance was identified. Based on these results, this study provides novel insights into the functional mechanisms of foxtail millet G proteins in salt-stress responses.

## 2. Materials and Methods

### 2.1. Genome-Wide Identification and Chromosomal Localization of G-Protein Genes in Foxtail Millet

To identify members of the G-protein gene family in foxtail millet, all protein sequences of foxtail millet were downloaded from the EnsemblPlants database (https://plants.ensembl.org/index.html, accessed on 20 March 2026). The reported G-protein sequences of *Arabidopsis thaliana* were used as reference sequences. A BLASTP search was performed using TBtools-II (v2.371) [28], with an E-value threshold of ≤1 × 10^−5^. To avoid missing potential G-protein family members, a hidden Markov model (HMM)-based search was further performed. The HMM profiles of Gα (PF00503), Gβ (PF00400) and Gγ (PF00631) were downloaded from the Pfam database (https://www.ebi.ac.uk/interpro/entry/pfam/#table, accessed on 20 March 2026) and used to search the foxtail millet protein database with a significance threshold of *p* < 0.001. Candidate sequences obtained from BLASTP and HMM searches were merged, and redundant sequences were removed. The conserved domains of all candidate proteins were further verified using SMART (http://smart.embl.de/, accessed on 21 March 2026) and the NCBI Conserved Domain Database CDD (https://www.ncbi.nlm.nih.gov/cdd/, accessed on 21 March 2026) [29,30,31]. Non-redundant genes containing the corresponding conserved domains were identified as members of the foxtail millet G-protein gene family and were renamed according to their homology with *Arabidopsis* G-protein genes. Chromosome length information of foxtail millet and the chromosomal positions of G-protein genes were obtained using TBtools-II (v2.371), and the distribution was subsequently visualized.

### 2.2. Analysis of Conserved Motifs, Gene Structures and Protein Features

The physicochemical properties of the identified foxtail millet G proteins, including amino acid length and instability index, were analyzed using TBtools-II (v2.371). Conserved motifs of the foxtail millet G-protein family were predicted using the MEME online program (https://meme-suite.org/meme/, accessed on 21 March 2026). The maximum number of motifs was set to 15, and all other parameters were kept at their default settings. The results were saved in MEME.xml format. The whole-genome sequence and GFF3 annotation file of foxtail millet were downloaded from the EnsemblPlants database (https://plants.ensembl.org/index.html, accessed on 21 March 2026). The conserved motif information, gene structure annotation and phylogenetic tree of the foxtail millet G-protein family were integrated and visualized using TBtools-II (v2.371).

### 2.3. Multiple Sequence Alignment and Phylogenetic Analysis

To clarify the evolutionary relationships among G-protein family members, the protein sequences of 12 foxtail millet, 8 *Arabidopsis thaliana*, 11 rice (*Oryza sativa*), 10 maize (*Zea mays*) and 9 sorghum (*Sorghum bicolor*) G-protein members were used for phylogenetic analysis. Multiple sequence alignment was performed using the ClustalW algorithm in MEGA 12 v12.0.11. Based on the alignment results, a phylogenetic tree was constructed using the neighbor-joining (NJ) method in MEGA 12. The resulting phylogenetic tree was further annotated and visualized using the iTOL online platform (https://itol.embl.de/, accessed on 21 March 2026) [32].

### 2.4. Synteny and Collinearity Analysis

The genome FASTA files and GFF3 annotation files of foxtail millet, *Arabidopsis thaliana*, rice, maize and wheat were downloaded from the EnsemblPlants database. Intraspecific and interspecific collinearity relationships were analyzed using the MCScanX module in TBtools-II (v2.371). The syntenic relationships of G-protein genes within the foxtail millet genome and between foxtail millet and other plant species were visualized using TBtools-II (v2.371).

### 2.5. Analysis of Cis-Acting Elements in Promoter Regions

The 2000 bp upstream sequences from the transcription start sites of foxtail millet G-protein genes were extracted using TBtools-II (v2.371) and submitted to the PlantCARE database (http://bioinformatics.psb.ugent.be/webtools/plantcare/html/, accessed on 21 March 2026) for prediction of *cis*-acting regulatory elements. *Cis*-acting elements associated with stress responses, hormone responses and light responses were retained for further analysis. The distribution of these promoter elements was visualized using TBtools-II (v2.371).

### 2.6. Plant Materials, Salt Treatment and RNA-Seq Analysis

Seeds of the foxtail millet cultivar Jingu 21 with uniform size, full grains and no visible damage were selected for the salt-stress experiment. The seeds were surface-sterilized with 5% sodium hypochlorite solution (Shanghai Aladdin Biochemical Technology Co., Ltd., Shanghai, China) for 10 min and then rinsed three times with distilled water. The sterilized seeds were placed in germination boxes lined with gauze and grown under controlled conditions at 25 °C, with an 8 h light/16 h dark photoperiod and 75% relative humidity. When the seedlings reached the two-leaf and one-heart stage, they were treated with a 200 mmol/L NaCl solution. Root samples were collected at 12 and 24 h after salt treatment; plants grown in a normal nutrient solution at 12 h and 24 h time points served as the corresponding control groups. Root samples (three biological replicates) were collected after one day of treatment. Total RNA was extracted using the ethanol precipitation method combined with the RNAprep Pure Plant Kit (Cat. No. DP441, TIANGEN BIOTECH (BEIJING) CO., LTD. Beijing, China), subsequently, reverse transcription was performed (Table S2). The concentration and quality of total RNA were evaluated using a Qubit 4.0 fluorometer (Thermo Fisher Scientific, Waltham, MA, USA) and a Qsep400 high-throughput bio-fragment analyzer (Advanced Instruments, Inc., Norwood, MA, USA), respectively. RNA-seq library construction and sequencing were performed by Metware Biotechnology Co., Ltd. (Wuhan, China).

Differential expression analysis was performed using DESeq2 (v1.49.6) [33,34]. Read counts for each gene were used as input data. The read count data were normalized, and statistical tests were performed based on the DESeq2 model. Multiple testing correction was conducted to calculate the false discovery rate (FDR). Genes with |log_2_FC| ≥ 1 and FDR ≤ 0.05 were identified as differentially expressed genes (DEGs) in each comparison.

## 3. Results

### 3.1. Identification, Chromosomal Localization and Physicochemical Properties of Foxtail Millet G-Protein Genes

**Figure 1:**
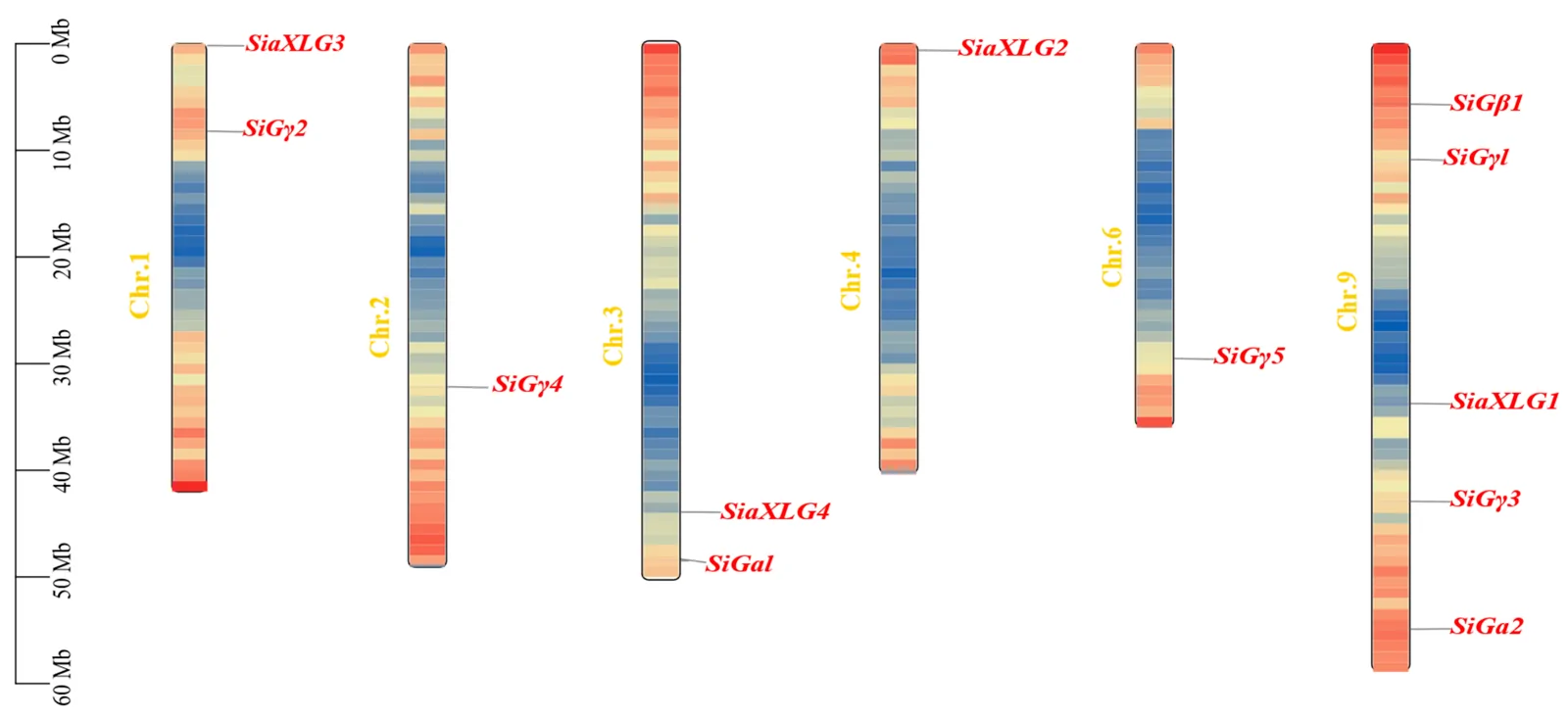
Chromosomal distribution of G-protein genes in *Setaria italica*.

A total of 12 G-protein genes were identified in the foxtail millet genome and used for further analysis. These genes included six Gα-related members, consisting of two canonical Gα genes and four extra-large G-protein genes, one Gβ gene and five Gγ genes. Chromosomal localization analysis showed that the 12 G-protein genes were unevenly distributed on chromosomes 1, 2, 3, 4, 6 and 9 (Figure 1). Chromosome 9 contained the largest number of G-protein genes, with five members (*SiGβ1*, *SiGγ1*, *SiαXLG1*, *SiGγ3*, and *SiGα2*), whereas the other chromosomes contained one or two genes.

The physicochemical properties of the identified foxtail millet G proteins were further analyzed (Table 1). The amino acid lengths of these proteins ranged from 97 to 954 aa, with SiGγ1 being the shortest and SiαXLG4 being the longest. Their predicted molecular weights ranged from 10,903.30 to 103,668.68 Da. SiGγ1 had the lowest molecular weight, whereas SiαXLG4 had the highest molecular weight. The theoretical isoelectric points ranged from 4.99 to 8.62. The instability index values of the 12 G proteins ranged from 31.29 to 83.45. Among them, only SiGβ1 was predicted to be a stable protein, whereas the remaining members were predicted to be unstable proteins. The aliphatic index ranged from 33.73 to 84.92. The grand average of hydropathicity values of all 12 proteins was negative, indicating that these foxtail millet G proteins are hydrophilic proteins.

### 3.2. Phylogenetic Analysis of G-Proteins from Different Plant Species

**Figure 2:**
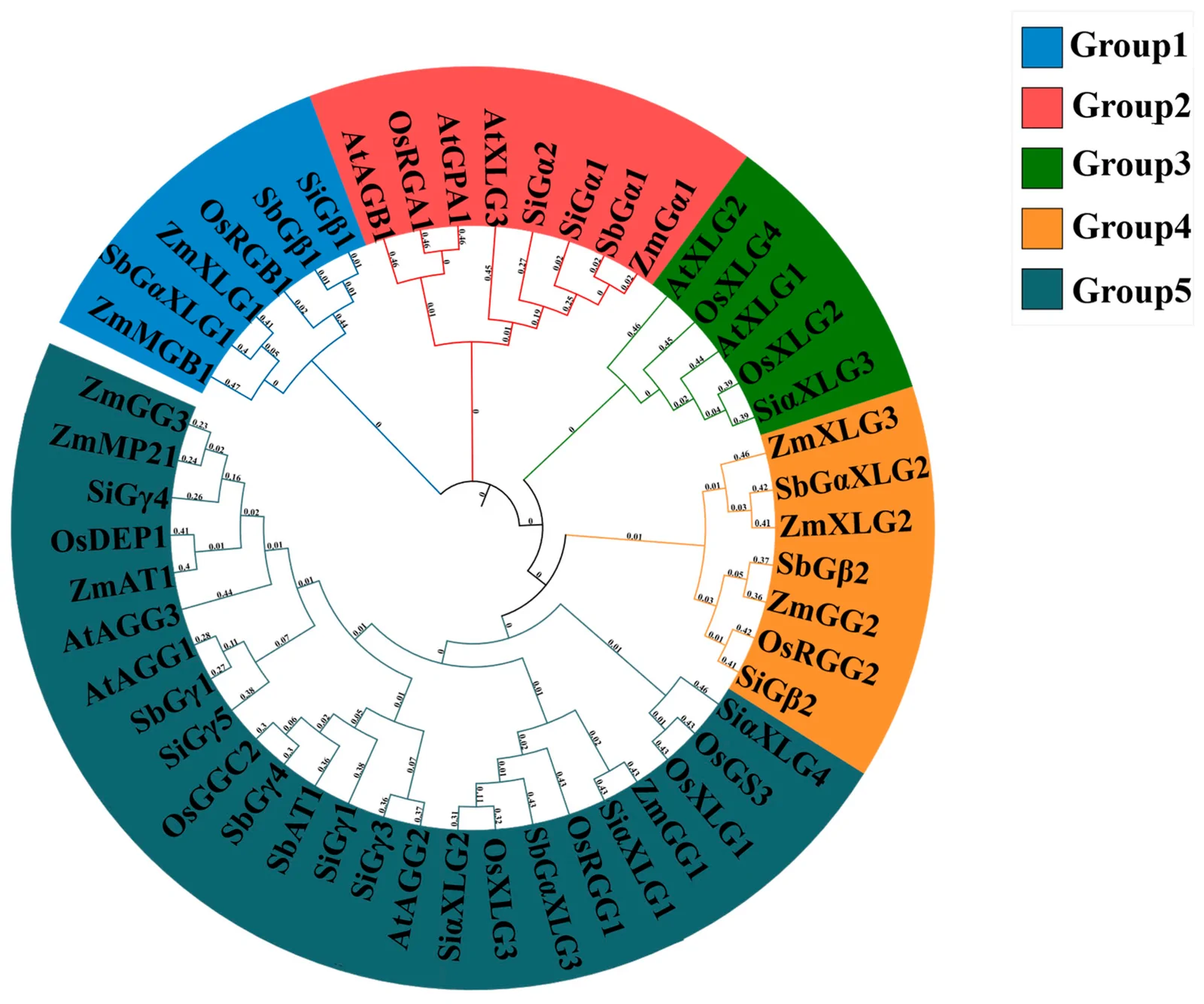
Phylogenetic tree of G proteins from *Setaria italica* (Si), *Arabidopsis thaliana* (At), *Oryza sativa* (Os), *Zea mays* (Zm) and *Sorghum bicolor* (Sb).

To investigate the evolutionary relationships of G-protein family members, a phylogenetic tree was constructed using G-protein sequences from foxtail millet, *Arabidopsis thaliana*, rice (*Oryza sativa*), maize (*Zea mays*) and sorghum (*Sorghum bicolor*) (Figure 2). Based on the phylogenetic relationships, these G-protein members were divided into five groups. Group 1 mainly contained Gβ subunits, including AtAGB1, OsRGB1, ZmMGB1 and SbGB1. These members clustered closely together, suggesting that Gβ proteins are highly conserved during plant evolution. Group 3 mainly consisted of XLG-type Gα-related proteins, including AtXLG1, AtXLG2, OsXLG2, OsXLG4 and SiαXLG3, indicating that XLG members may have undergone lineage-specific differentiation during evolution. Members in this group may be involved in the regulation of plant growth, development and stress responses. Group 4 contained several Gγ subunits, such as ZmGG2 and OsRGG2, which are known to function together with Gβ proteins as Gβγ dimers. Group 5 contained the largest number of G-protein members, suggesting that these genes may share common ancestral origins and may have expanded through gene duplication during evolution. Overall, the phylogenetic results indicated both conservation and divergence of G-protein family members among different plant species.

### 3.3. Conserved Motifs, Conserved Domains and Gene Structures of Foxtail Millet G-Protein Family Members

**Figure 3:**
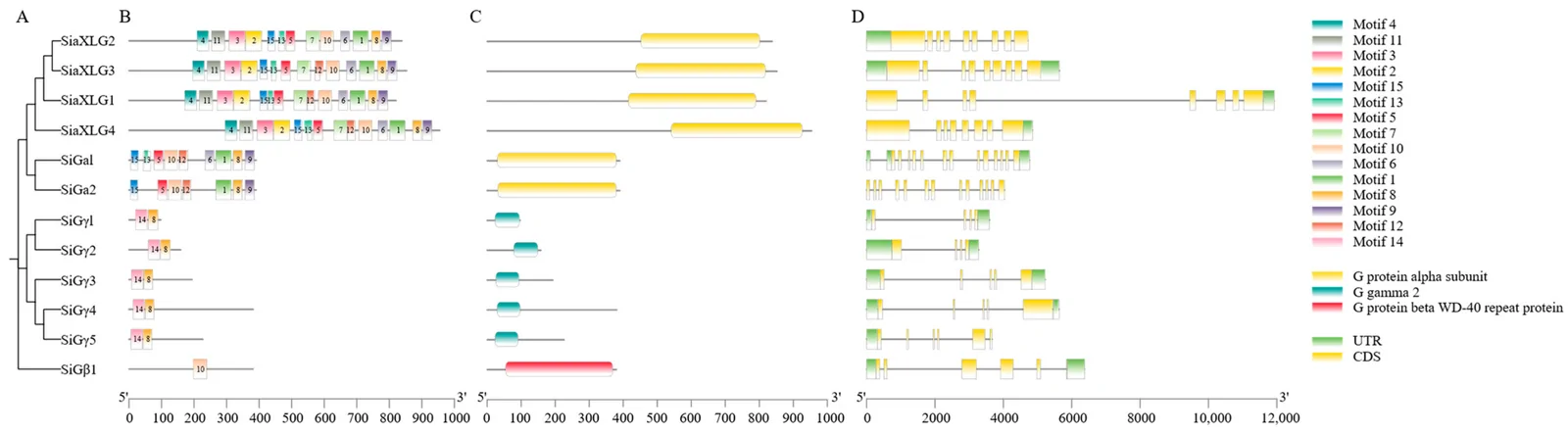
Conserved motif, conserved domain and gene structure analyses of G-protein gene family members in *Setaria italica*. (**A**,**B**) Conserved motif distribution. (**C**) Conserved domain organization. (**D**) Exon–intron structures.

To further characterize the structural diversity of foxtail millet G proteins, conserved motifs were analyzed and named Motif 1 to Motif 15 (Figure 3B). Members belonging to the same subunit type generally shared similar motif compositions, indicating that G proteins within the same subfamily are relatively conserved. Fourteen motifs were detected in SiαXLG1–SiαXLG4 and showed relatively conserved distributions, suggesting that these motifs may contribute to the functional conservation of XLG-type Gα-related proteins. Only one conserved motif was detected in SiGβ1, indicating a distinct motif composition compared with the Gα- and Gγ-related members.

Conserved domain analysis showed that SiαXLG1–SiαXLG4 and SiGα1–SiGα2 contained typical Gα-related domains, which are responsible for GTP/GDP binding and hydrolysis and function as molecular switches in signal transduction (Figure 3C). SiGγ1–SiGγ5 contained Gγ-related domains, which are characteristic of Gγ proteins and are involved in the formation of Gβγ dimers, membrane localization and signal regulation. SiGβ1 contained a WD40 repeat domain, which is the typical structural feature of Gβ proteins and forms a β-propeller structure that provides a platform for interactions with Gα, Gγ and downstream effector proteins. The distribution of conserved domains was largely consistent with the phylogenetic classification, suggesting functional conservation within the same subfamily and functional differentiation among different subfamilies.

Gene structure analysis showed that the 12 foxtail millet G-protein genes differed in exon–intron organization (Figure 3D). The number of exons ranged from 4 to 14, and the number of introns ranged from 3 to 13. SiGα1 contained the largest number of exons and introns, with 14 exons and 13 introns, whereas the other genes contained 4–13 exons and 3–12 introns. Genes belonging to the same G-protein subunit type generally showed similar exon–intron structures, which was consistent with the phylogenetic analysis and further supported the structural conservation of each subfamily. Differences in gene structure may reflect the evolutionary diversification of the G-protein gene family.

### 3.4. Collinearity Analysis of the Foxtail Millet G-Protein Gene Family

**Figure 4:**
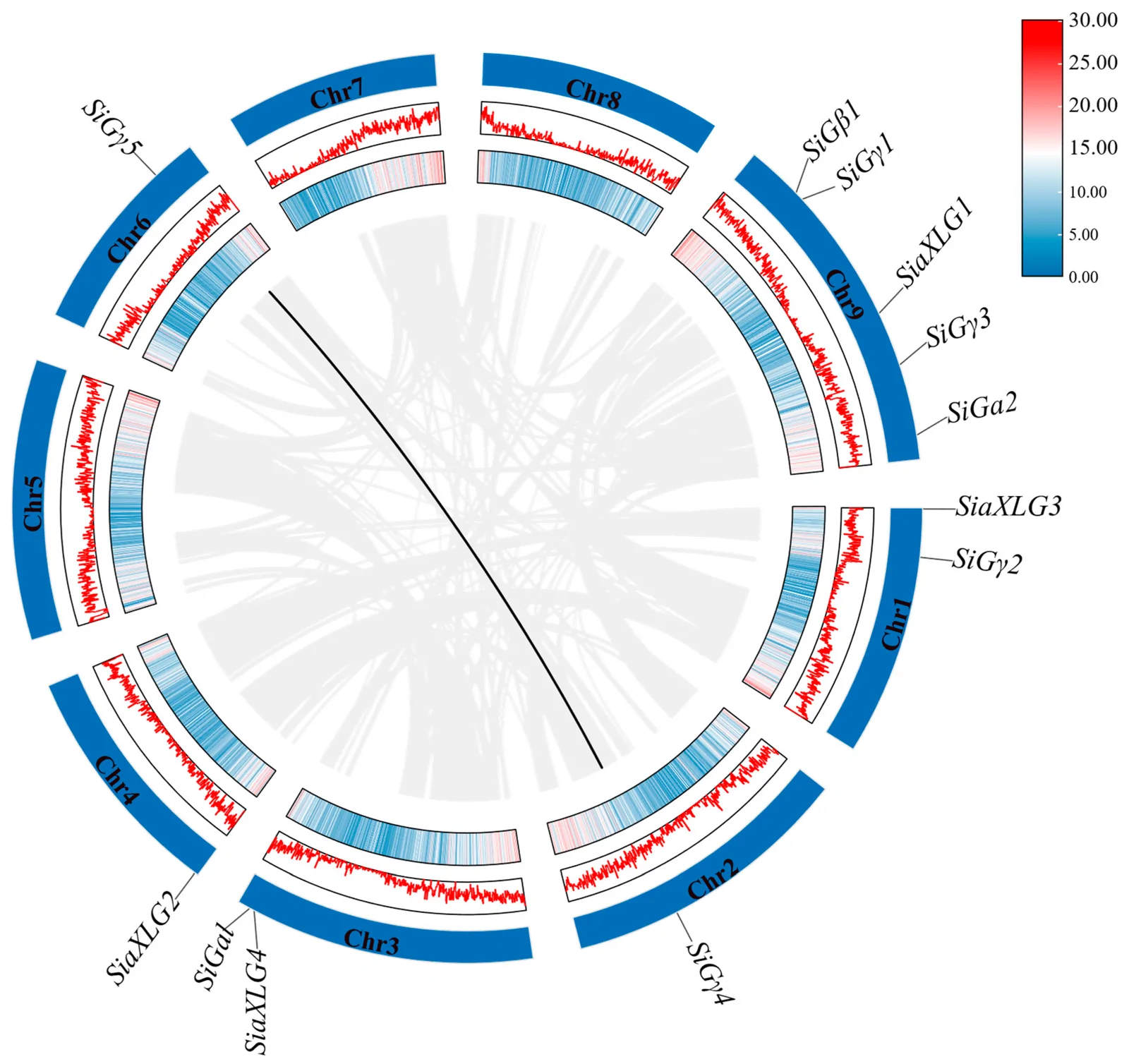
Intraspecific collinearity of G-protein genes in *Setaria italica.* The collinear gene pairs are marked by black lines. The red-to-blue gradient regions on the chromosomes represent gene density (red indicates dense gene distribution, while blue indicates sparse distribution), and the color scale on the right clarifies the density value range (0.00–30.00).

To investigate duplication events and evolutionary relationships within the foxtail millet G-protein gene family, intraspecific collinearity analysis was performed. The results showed that one segmentally duplicated gene pair was identified among the 12 foxtail millet G-protein genes, namely SiGγ4 on chromosome 2 and SiGγ5 on chromosome 6 (Figure 4). This result suggests that segmental duplication may have contributed to the expansion of the Gγ subfamily in foxtail millet.

**Figure 5:**
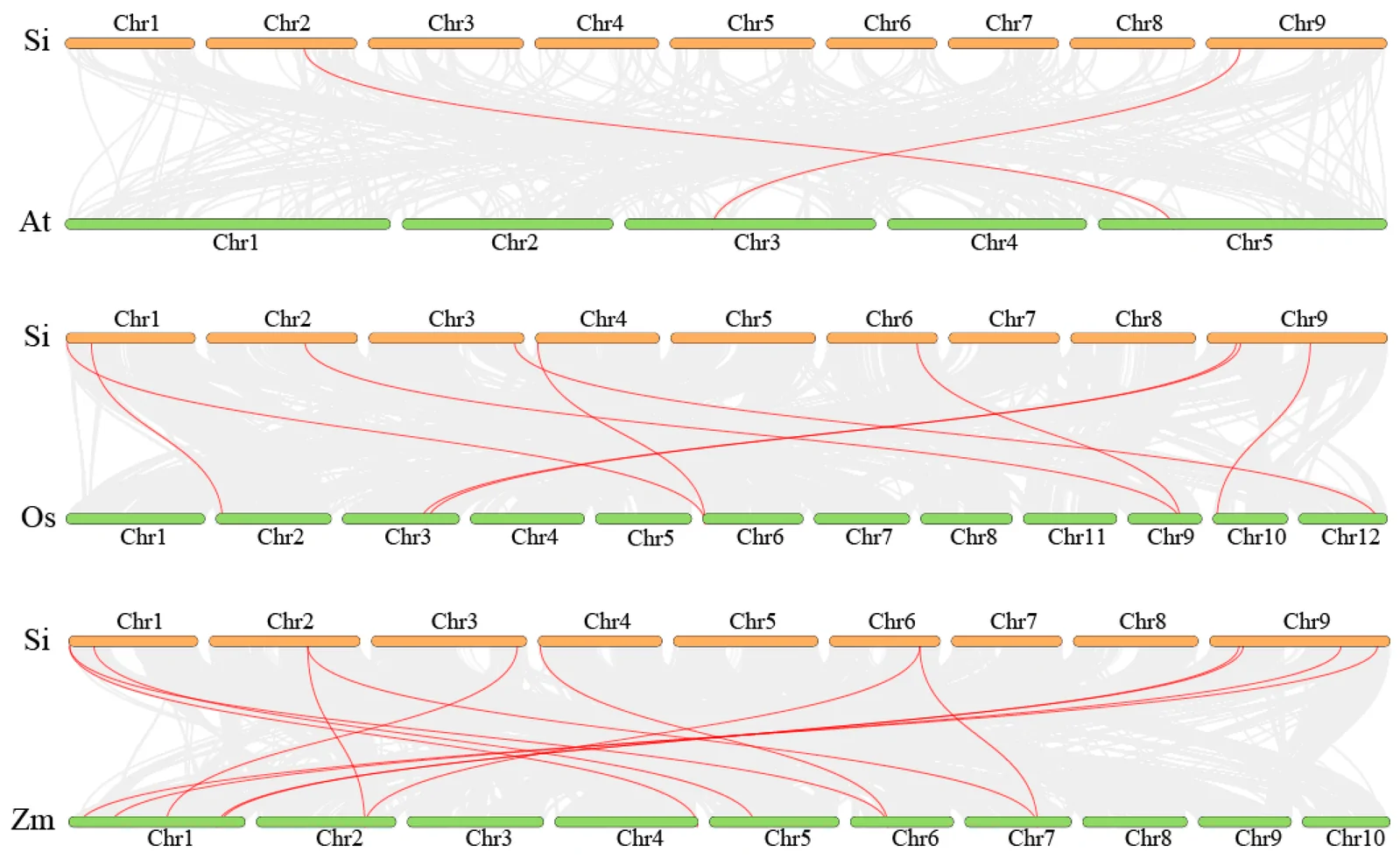
Interspecific collinearity of G-protein genes among *Setaria italica* (Si), *Arabidopsis thaliana* (At), *Oryza sativa* (Os) and *Zea mays* (Zm). Red lines indicate collinear gene pairs across distinct species.

Interspecific collinearity analysis was further conducted between foxtail millet and *Arabidopsis thaliana*, rice and maize (Figure 5). Several foxtail millet G-protein genes showed syntenic relationships with homologous genes in other species. For example, some G-protein genes located on chromosomes 2 and 9 of foxtail millet showed collinearity with G-protein genes located on chromosomes 5 and 3 of *Arabidopsis thaliana*, respectively. Extensive collinearity was also observed between foxtail millet G-protein genes and their homologs in rice and maize. These conserved syntenic relationships indicate that G-protein family members are evolutionarily conserved among different plant species and may have similar biological functions.

### 3.5. Cis-Acting Elements in the Promoter Regions of Foxtail Millet G-Protein Genes

**Figure 6:**
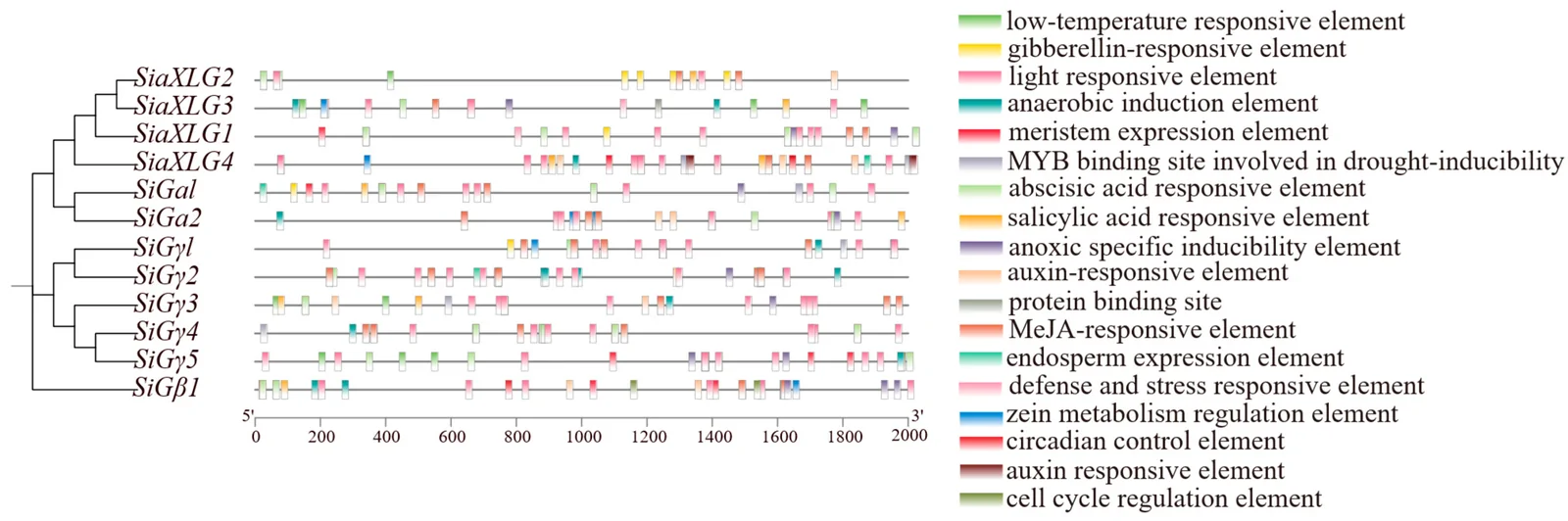
*Cis*-acting element analysis of promoter regions of foxtail millet G-protein genes.

To explore the potential transcriptional regulatory mechanisms of foxtail millet G-protein genes, *cis*-acting elements in their promoter regions were analyzed. A total of 18 representative *cis*-acting elements were selected for further analysis (Figure 6). These elements were mainly associated with light responses, hormone responses, biotic stress responses and abiotic stress responses.

Hormone-responsive elements included elements related to gibberellin (GA), abscisic acid (ABA), methyl jasmonate (MeJA), auxin and salicylic acid (SA). Almost all foxtail millet G-protein genes contained MeJA-responsive elements, especially *SiαXLG2*, *SiGγ3* and *SiGβ1*, suggesting that these genes may be involved in jasmonate-mediated defense responses and growth regulation. ABA-responsive elements were also present in most G-protein genes and were particularly abundant in *SiGγ5*. Because ABA is a key hormone involved in plant responses to abiotic stresses, these results suggest that some foxtail millet G-protein genes may participate in ABA-mediated stress signaling pathways.

GA-responsive elements were mainly detected in *SiαXLG2*, *SiGα1*, *SiGγ3* and *SiGβ1*, suggesting that these genes may be involved in GA-regulated developmental processes, such as seed germination, stem elongation and flowering. SA-responsive elements were mainly distributed in *SiαXLG2*, *SiαXLG4*, *SiGγ2* and *SiGβ1*, indicating that these genes may participate in SA-mediated biotic stress responses. Auxin-responsive elements were identified in *SiαXLG2*, *SiGα2*, *SiGγ3* and *SiGβ1*, suggesting potential roles of these genes in auxin-mediated regulation of cell division, cell elongation and organ development.

In addition to hormone-responsive elements, multiple stress- and development-related *cis*-acting elements were detected. *SiGγ5* contained abundant low-temperature-responsive elements, suggesting that this gene may be involved in cold-stress responses in foxtail millet. Drought-inducible MYB-binding sites were detected in *SiαXLG3*, *SiαXLG4*, *SiGγ2*, *SiGγ3* and *SiGβ1*, indicating that the G-protein gene family may be broadly involved in drought-stress responses. Meristem-expression-related elements were found in *SiαXLG1*, *SiαXLG4*, *SiGγ4* and *SiGβ1*, suggesting possible roles in meristem development and organ formation. Endosperm-expression-related elements were detected in *SiGγ2*, *SiGγ4* and *SiGγ5*, indicating potential involvement in seed development and nutrient accumulation. Circadian rhythm-related elements were present in *SiαXLG4*, *SiGγ5* and *SiGβ1*, suggesting that these genes may also participate in circadian regulation.

Overall, the promoter regions of foxtail millet G-protein genes contained diverse *cis*-acting regulatory elements, indicating that these genes may be widely involved in plant growth, development, hormone signaling and stress responses.

### 3.6. Transcriptome Analysis Under Salt Stress

To investigate the expression responses of foxtail millet G-protein genes under salt stress, RNA-seq was performed on root samples collected from the control and salt-treated seedlings. After quality filtering, an average of 59,733,973 clean reads was obtained per sample (Table S1). The Q20 values of all samples were greater than 99%, and the Q30 values were greater than 97%. The GC content ranged from 53.98% to 55.26%. These results indicated that the RNA-seq data were of high quality and suitable for subsequent transcriptome analysis.

**Figure 7:**
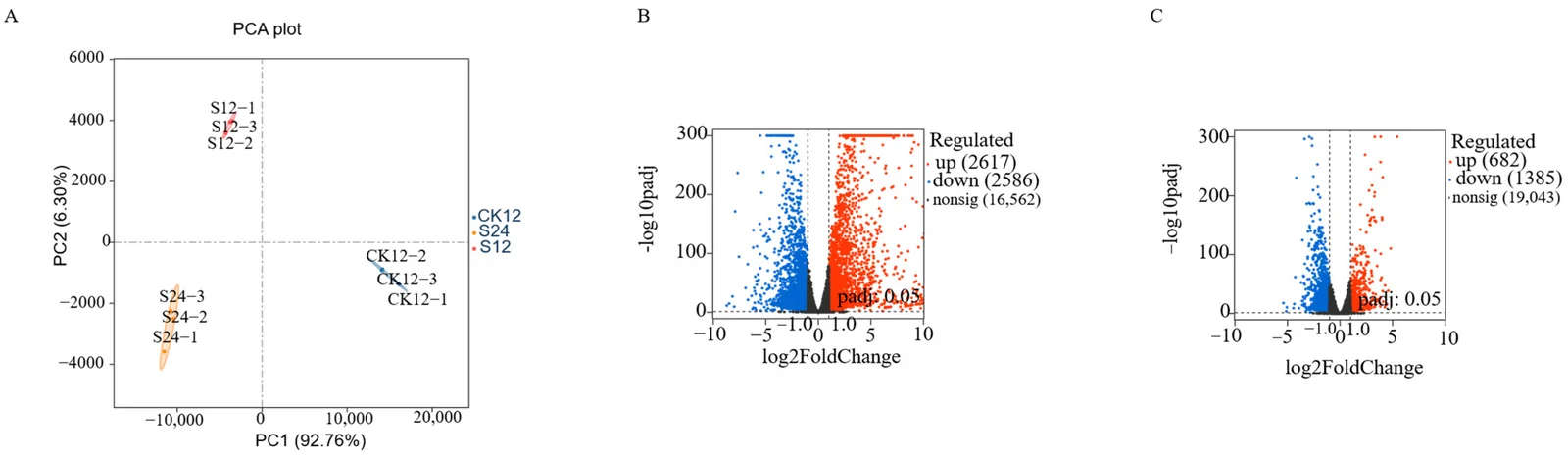
Transcriptomic analysis of foxtail millet under salt-stress treatment. (**A**) PCA. S, salt stress; CK, control. (**B**,**C**) Volcano plots of differentially expressed genes across three salt stress-related comparisons ((**B**) CK12 vs. S12; (**C**) S12 vs. S24). The horizontal dashed line represents padj = 0.05 (adjusted *p*-value based on FDR correction).

Principal component analysis (PCA) was performed to evaluate the overall transcriptional variation among samples (Figure 7A). The samples from different treatment groups were clearly separated at the transcriptome level, indicating that the first two principal components effectively captured the major variation in gene expression. Along PC1, the salt-treated samples (S12 and S24) were clearly separated from the control samples (CK12). Salt-treated samples were mainly distributed on the negative side of PC1, whereas control samples were distributed on the positive side, suggesting that salt treatment was the primary factor driving transcriptomic differences among samples. Along PC2, the S12 and S24 samples were further separated, indicating that treatment duration also contributed to transcriptome variation under salt stress. In addition, biological replicates within each treatment group clustered closely together, and the within-group distances were smaller than the between-group distances. These results demonstrated good biological reproducibility and confirmed that the RNA-seq data were reliable for differential expression analysis.

To further explore the molecular responses of foxtail millet to salt stress, differentially expressed genes (DEGs) were identified among different comparison groups and visualized using volcano plots. In the CK12 vs. S12 comparison, a total of 5203 DEGs were detected, including 2617 up-regulated and 2586 down-regulated genes, while 16,562 genes showed no significant differential expression (Figure 7B). In the S12 vs. S24 comparison, 2067 DEGs were identified, including 682 up-regulated and 1385 down-regulated genes, while 19,043 genes were not significantly differentially expressed (Figure 7C). Overall, the CK12 vs. S12 comparison showed a larger number of DEGs with a relatively balanced distribution of up- and down-regulated genes, indicating that salt stress induced broad bidirectional transcriptional regulation at the early response stage. In contrast, fewer DEGs were detected in the S12 vs. S24 comparison, and down-regulated genes were predominant, suggesting that prolonged salt treatment mainly resulted in transcriptional repression of a subset of genes.

**Figure 8:**
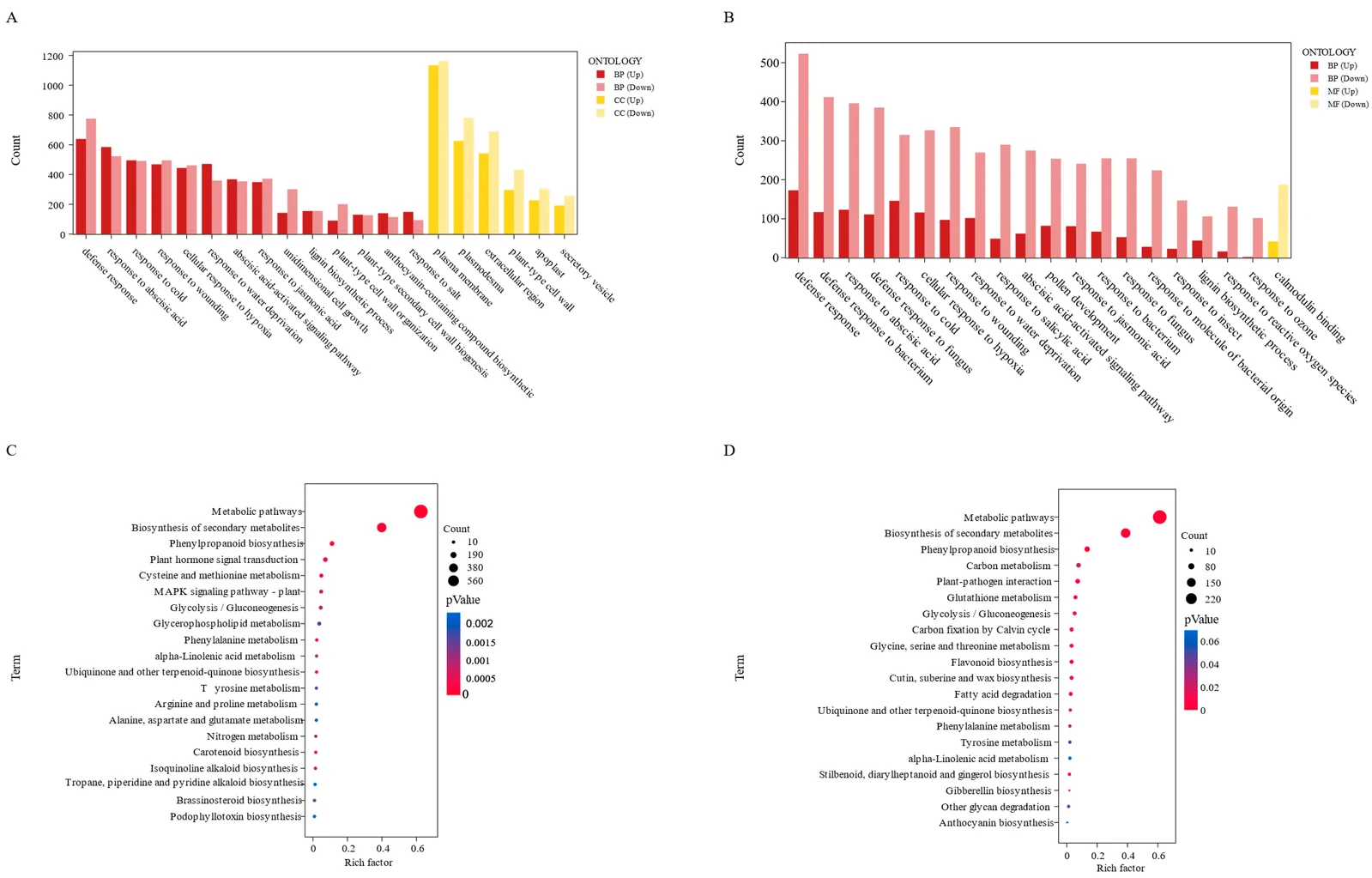
GO and KEGG analysis of two comparison groups. (**A**,**B**) GO enrichment analysis of the comparison group CK vs. S12. (**C**,**D**) KEGG enrichment analysis of the comparison group S12 vs. S24 (BP: biological process; CC: cellular component; MF: molecular function).

GO functional enrichment and KEGG pathway enrichment analyses were separately performed on differentially expressed genes (DEGs) from the two comparison groups (Figure 8A–D). For DEGs derived from CK vs. S12 (Figure 8A), biological process (BP) terms were markedly enriched in defense responses, stress responses, and plant cell wall biosynthesis, while cellular component (CC) terms were predominantly associated with the plasma membrane, extracellular region and cell wall structures. For DEGs of S12 vs. S24 (Figure 8B), up-regulated BP entries were still dominated by diverse biotic and abiotic stress responses, and calcium-ion-binding activity was significantly enriched in molecular function (MF). Stress-related biological processes were abundantly enriched in both comparison groups, and the enrichment magnitude of genes related to oxidative stress and lignin biosynthesis increased with prolonged salt-stress treatment.

The KEGG bubble plots of both comparisons revealed that metabolic pathways and biosynthesis of secondary metabolites exhibited the highest enrichment. For CK vs. S12 (Figure 8C), phenylpropanoid biosynthesis, plant hormone signal transduction, and the plant MAPK signaling pathway were prominently enriched. In S12 vs. S24 (Figure 8D), besides secondary metabolic pathways, salt-tolerant pathways including flavonoid biosynthesis, cutin, suberine and wax biosynthesis, and glutathione metabolism showed significant enrichment. The enrichment effects of antioxidant and cell wall modification metabolic pathways were strengthened as salt-stress duration extended.

### 3.7. Expression Profiles and Candidate Gene Analysis of G-Protein Family Members Under Salt Stress

**Figure 9:**
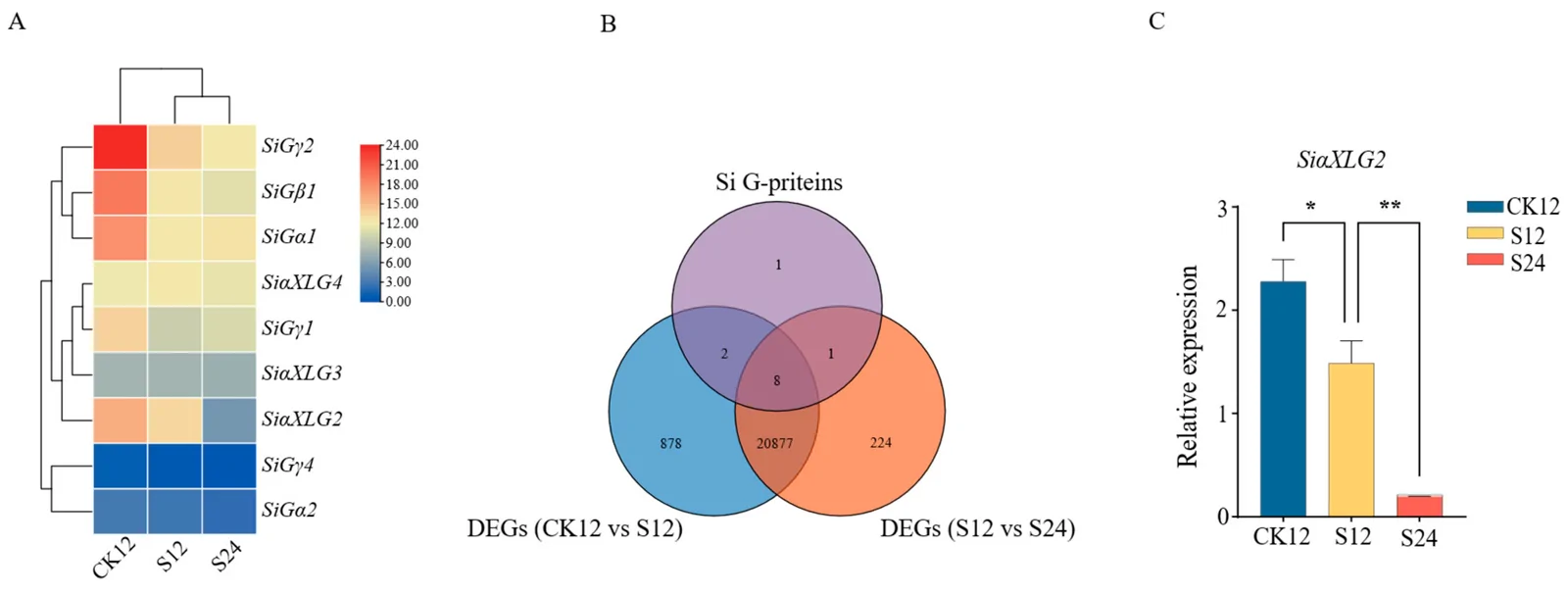
Expression patterns of G-protein family members in foxtail millet and correlation analysis of candidate genes. (**A**) Expression profiles of foxtail millet G proteins under salt stress. (**B**) Venn diagram of transcriptomic DEGs with G proteins in foxtail millet. (**C**) Expression analysis of *SiαXLG2* under normal and salt-stress conditions. * *p*-value ≤ 0.1, ** *p*-value ≤ 0.01.

To explore the expression characteristics of foxtail millet G-protein genes under salt stress, the expression profiles of the identified G-protein family members were visualized using the salt-stress RNA-seq data (Figure 9A). By integrating the G-protein gene list with the DEG results, nine G-protein genes were found to be differentially expressed in at least one comparison group (Figure 9B). Among the differentially expressed G-protein genes, SiαXLG2 showed a clear salt-responsive expression pattern. RT-qPCR assays (Figure 9C) revealed a gradual reduction in its expression from CK12 to S12 and S24; these differences were statistically significant, and the expression pattern matched the transcriptomic profiles (Figure 9A). This expression pattern suggests that *SiαXLG2* may be involved in the transcriptional regulation of salt-stress responses in foxtail millet. Therefore, SiαXLG2 was considered a key candidate gene for further functional analysis of G-protein-mediated salt-stress signaling. Further validation can be performed via gene-editing technology in future studies.

## 4. Discussion

G proteins are ubiquitous signal transduction molecules in eukaryotes and play important roles in regulating diverse biological processes in plants. As relatively conserved signaling components, heterotrimeric G proteins participate in plant growth, development, hormone signaling and environmental stress responses [35]. In the present study, 12 G-protein genes were identified in the foxtail millet genome, a number higher than that reported in *Arabidopsis thaliana* and rice [21,36]. This difference may reflect lineage-specific expansion and evolutionary diversification of G-protein family members among plant species. The phylogenetic analysis supported the classification of foxtail millet G proteins into distinct subunit types and evolutionary groups. In addition, gene structure and conserved domain analyses showed that members belonging to the same subunit type generally shared similar structural features, indicating functional conservation within each subfamily. Collinearity analysis identified a duplicated G-protein gene pair in foxtail millet, suggesting that gene duplication may have contributed to the evolution and expansion of this gene family. Functional divergence after duplication may have facilitated the adaptation of different plant species to diverse developmental and environmental conditions [37].

Promoter *cis*-acting element analysis provides useful clues for predicting the potential regulatory patterns of gene family members. In this study, all 12 foxtail millet G-protein genes contained at least one light-responsive element, suggesting that these genes may be regulated by light-associated signaling pathways. In addition, multiple hormone-responsive elements were identified in the promoter regions of foxtail millet G-protein genes, such as *SiaXLG1*, *SiaXLG2* and *SiGγ2*, indicating their potential involvement in hormone-mediated growth and developmental regulation. This finding is consistent with previous studies showing that loss or gain of G-protein function in *Arabidopsis* and rice can cause developmental and physiological changes [38,39,40,41]. Furthermore, different foxtail millet G-protein genes contained distinct stress-responsive elements, including elements associated with abiotic and biotic stress responses, such as *SiαXLG2*, *SiGγ1* and *SiGγ2*. These results suggest that foxtail millet G-protein genes may not only participate in growth and developmental processes but also contribute to responses to environmental stresses. Similar roles have been reported for rice G-protein genes under drought and salt stress and for *Arabidopsis* G-protein genes in pathogen responses [42,43,44]. Therefore, the promoter element composition of foxtail millet G-protein genes supports their potential functional diversification in response to different environmental signals.

RNA-seq is an effective approach for comprehensively characterizing transcriptome-wide gene expression changes under specific biological conditions. It has been widely used to identify differentially expressed genes and to reveal molecular mechanisms underlying plant stress tolerance [45,46,47,48]. In this study, RNA-seq analysis was performed using foxtail millet samples collected under different salt-stress treatments and time points. The sequencing quality, global expression variation, DEG profiles and expression patterns of G-protein family members were systematically analyzed. These results provide a transcriptomic basis for further understanding the molecular mechanisms of salt tolerance in foxtail millet.

The identification and analysis of differentially expressed genes are essential for elucidating the molecular response of foxtail millet to salt stress. In the CK12 vs. S12 comparison, 5203 DEGs were identified, including similar numbers of up- and down-regulated genes. This result indicates that salt stress induced extensive bidirectional transcriptional regulation in foxtail millet. The up-regulated genes may include positive regulators involved in salt-stress adaptation, whereas the down-regulated genes may be associated with growth-related or stress-sensitive processes that are suppressed under stress conditions. This pattern is consistent with previous transcriptome studies in crops such as wheat and maize, in which abiotic stress broadly reshaped gene expression profiles [49]. The large number of DEGs further suggests that salt stress markedly alters the physiological and metabolic status of foxtail millet, potentially through the activation or repression of multiple pathways related to osmotic adjustment [50], reactive oxygen species scavenging [51], ion homeostasis [52] and other stress-responsive processes.

In the S12 vs. S24 comparison, the number of DEGs was smaller than that in the CK12 vs. S12 comparison, and down-regulated genes were predominant. This pattern suggests that the intensity of transcriptional reprogramming decreased from 12 h to 24 h after salt treatment. One possible explanation is that foxtail millet rapidly initiates stress-response programs at the early stage of salt stress, leading to large-scale changes in gene expression. As the stress duration extends to 24 h, the plants may gradually adjust to the salt-stress environment, and transcriptomic regulation may shift toward maintaining cellular homeostasis rather than activating a large number of additional genes. This dynamic pattern is consistent with the general process of rapid stress response followed by adaptive adjustment in plants [53] and provides a basis for further investigating time-dependent regulatory mechanisms under salt stress.

Previous studies have shown that G-protein signaling is involved in plant responses to osmotic and salt stresses. Osmotic stress caused by high salinity can affect cell proliferation by modulating Gα-mediated signaling, and Gα loss-of-function mutations can alter sodium-toxicity-induced leaf senescence and growth inhibition under osmotic stress [54]. In the present study, hierarchical clustering of the 12 foxtail millet G-protein genes showed clear separation between control and salt-treated samples, indicating that salt stress had a stable regulatory effect on the expression of G-protein family members. Among these genes, *SiαXLG2* showed high expression in the control group but was significantly down-regulated under salt treatment. This expression pattern suggests that *SiαXLG2* may act as a salt-responsive G-protein gene in foxtail millet. Its repression under salt stress may be associated with the regulation of downstream stress-response pathways. However, further functional validation, such as qRT-PCR verification, gene overexpression, gene knockout or protein interaction analysis, is required to clarify its precise role in salt-stress tolerance.

Overall, the expression patterns of foxtail millet G-protein genes under salt stress revealed functional differentiation among family members. The identification of salt-responsive G-protein genes, particularly *SiαXLG2*, provides candidate gene resources for further dissecting G-protein-mediated salt-stress signaling pathways and for exploring key genes associated with salt tolerance in foxtail millet.

## 5. Conclusions

In this study, 12 G-protein family members were identified in foxtail millet and classified into three subunit types, including Gα-related, Gβ and Gγ members. Promoter analysis revealed that foxtail millet G-protein genes contain multiple *cis*-acting elements associated with light responses, hormone responses, biotic stress and abiotic stress, indicating their potential involvement in diverse regulatory processes. Transcriptome analysis under salt stress identified nine differentially expressed G-protein genes. Among them, *SiαXLG2* showed a significant salt-responsive expression pattern and was identified as a key candidate gene potentially involved in salt-stress signaling. These findings provide a foundation for further functional characterization of G-protein genes and offer candidate gene resources for improving salt tolerance in foxtail millet. Further functional verification of this gene can be carried out via gene editing [55] technology in future studies.

## Figures and Tables

**Table 1:** Physicochemical properties of the G-protein gene family in *Setaria italica*.

Gene Name	Sequence ID	Number of Amino Acids	Molecular Weight	Theoretical pI	Instability Index	Aliphatic Index	Grand Average of Hydropathicity
SiGα1	Seita.3G382500.1.v2.2	390	45,198.52	6.15	40.76	81.46	−0.522
SiGα2	Seita.9G519700.1.v2.2	390	45,571.21	6.38	40.93	84.92	−0.445
SiαXLG1	Seita.9G295300.1.v2.2	820	90,915.51	6.73	49.34	81.41	−0.339
SiαXLG2	Seita.4G009500.1.v2.2	838	93,369.07	5.56	54.85	73.27	−0.521
SiαXLG3	Seita.1G002600.1.v2.2	852	97,026.87	5.31	51.25	69.94	−0.586
SiαXLG4	Seita.3G373200.1.v2.2	954	103,668.68	5.71	59.83	75.45	−0.396
SiGβ1	Seita.9G145400.1.v2.2	380	41,700.74	7.15	31.29	75.42	−0.3
SiGγ1	Seita.9G162300.1.v2.2	97	10,903.3	4.99	62.93	72.47	−0.646
SiGγ2	Seita.1G093100.1.v2.2	158	17,784.26	5.16	72.91	44.49	−1.346
SiGγ3	Seita.9G369300.1.v2.2	193	20,538.11	8.58	69.9	55.23	−0.025
SiGγ4	Seita.2G219800.1.v2.2	381	40,697.89	8.39	83.45	33.73	−0.085
SiGγ5	Seita.6G171500.1.v2.2	226	23,871.23	8.62	80.36	47.52	−0.005

## Data Availability

The datasets presented in this study can be found in online repositories. The names of the repository/repositories and accession number(s) can be found below: https://submit.ncbi.nlm.nih.gov/subs/sra/ (accessed on 1 March 2026), PRJNA1483744.

## Supplementary Materials

The following supporting information can be downloaded at https://www.mdpi.com/article/10.3390/biology15161339/s1, Table S1: Statistics of sequencing data quality. Table S2: Primer sequences used in this study.
